# Revised Modelling of the Addition of Synchronous Chemotherapy to Radiotherapy in Squamous Cell Carcinoma of the Head and Neck—A Low *α/β*?

**DOI:** 10.3390/medicines5020054

**Published:** 2018-06-13

**Authors:** James Best, Charles Fong, Helen Benghiat, Hisham Mehanna, John Glaholm, Andrew Hartley

**Affiliations:** Hall-Edwards Radiotherapy Research Group, Queen Elizabeth Hospital, Birmingham B15 2TH, UK; james.best@uhb.nhs.uk (J.B.); charles.fong@uhb.nhs.uk (C.F.); Helen.Benghiat@uhb.nhs.uk (H.B.); hisham.mehanna@uhb.nhs.uk (H.M.); john.glaholm@nhs.net (J.G.)

**Keywords:** head and neck cancer, chemoradiation, linear quadratic equation

## Abstract

**Background:** The effect of synchronous chemotherapy in squamous cell carcinoma of the head and neck (SCCHN) has been modelled as additional Biologically Effective Dose (BED) or as a prolonged tumour cell turnover time during accelerated repopulation. Such models may not accurately predict the local control seen when hypofractionated accelerated radiotherapy is used with synchronous chemotherapy. **Methods:** For the purposes of this study three isoeffect relationships were assumed: Firstly, from the RTOG 0129 trial, synchronous cisplatin chemotherapy with 70 Gy in 35 fractions over 46 days results in equivalent local control to synchronous cisplatin chemotherapy with 36 Gy in 18# followed by 36 Gy in 24# (2# per day) over a total of 39 days. Secondly, in line with primary local control outcomes from the PET-Neck study, synchronous cisplatin chemotherapy with 70 Gy in 35# over 46 days results in equivalent local control to synchronous cisplatin chemotherapy delivered with 65 Gy in 30# over 39 days. Thirdly, from meta-analysis data, 70 Gy in 35# over 46 days with synchronous cisplatin results in equivalent local control to 84 Gy in 70# over 46 days delivered without synchronous chemotherapy. Using the linear quadratic equation the above isoeffect relationships were expressed algebraically to determine values of *α*, *α/β*, and *k* for SCCHN when treated with synchronous cisplatin using standard parameters for the radiotherapy alone schedule (*α* = 0.3 Gy^−1^, *α/β* = 10 Gy, and *k* = 0.42 Gy_10_day^−1^). **Results:** The values derived for *α/β*, *α* and *k* were 2 Gy, 0.20 and 0.21 Gy^−1^, and 0.65 and 0.71 Gy_2_day^−1^. **Conclusions:** Within the limitations of the assumptions made, this model suggests that accelerated repopulation may remain a significant factor when synchronous chemotherapy is delivered with radiotherapy in SCCHN. The finding of a low *α/β* for SCCHN treated with cisplatin suggests a greater tumour susceptibility to increasing dose per fraction and underlines the importance of the completion of randomized trials examining the role of hypofractionated acceleration in SCCHN.

## 1. Introduction

Twenty Eight years have passed since the linear quadratic (LQ) equation was proposed as a model for use in comparing radiotherapy fractionation schedules and 10 years since the late Professor Jack Fowler published his overviews of the optimal timing for radiotherapy alone schedules in squamous cell carcinoma of the head and head and neck cancer (SCCHN) [1,2,3,4]. Over this time, the LQ equation has been employed to predict potential benefits from proposed new fractionation schedules. However, the LQ equation with standard modification for repopulation fails to model the lack of benefit gained from accelerated fractionation with synchronous chemotherapy when this is compared with synchronous chemotherapy delivered with standard fractionation in SCCHN [5,6].

Five years ago a suppression of accelerated repopulation by synchronous chemotherapy as an explanation for the lack of benefit from accelerated fractionation in this setting was proposed [7]. This adjusted model gives reasonable predictions for many historic trials.

A recent meta-analysis has shown that roughly equivalent improvements in survival can be achieved either with synchronous chemotherapy and standard fractionation (70 Gy in 35# delivered over 46 days) or with hyperfractionated dose escalated radiotherapy alone (e.g., 84 Gy in 70# delivered over 46 days) [8]. These two schedules are yet to be directly compared in a randomized study. If the biologically effective dose for tumour (*tBED*) is calculated for both schedules using Equation 1 (*α* = 0.3 Gy^−1^; *β* = 0.03 Gy^−2^; *T_k_* = 21 days; *T_p_* = 3 days for radiotherapy alone or 10 days with synchronous chemotherapy) BEDs of 78 Gy_10_ and 75 Gy_10_, respectively, are obtained [3].

(1) $$tBED=D\left[1+\left(\frac{d}{\frac{\alpha }{\beta }}\right)\right]-\left[\frac{ln2}{\alpha }\times \frac{\left(T-T_{k}\right)}{T_{p}}\right]$$

where *tBED* = biologically effective dose for tumour (Gy*_α/β_*), *D* = total dose (Gy), *d* = dose per fraction (Gy), *α* = linear component of cell kill (Gy^−1^), *β* = quadratic component cell kill (Gy^−2^) *T* = overall treatment time (days), *T_k_* = kick off time (days) (modelled time of commencement of accelerated repopulation), and *T_p_* = clonogen doubling time (days).

If the log cell kill is calculated using Equation (2) log cell kills of 10.2 and 9.7 are obtained.

(2) $$log_{10\;}cell\;kill=\frac{tBED*\alpha }{\left(\frac{log_{10}}{log_{e}}\right)}$$

where *tBED* = biologically effective dose for tumour (Gy*_α/β_*) and *α* = linear component of cell kill (Gy^−1^).

If the dose gradient or ratio of % change in local control to % change in *tBED* at this dose level is assumed from previous modelling to be 1.2, a difference in local control of approximately 5% might be expected [9]. Such a difference would require a very large randomized study to detect. In addition, in certain head and neck sub groups the dose gradient may be significantly less than 1.2 within this dose range, thus any difference between these approaches would be even more difficult to detect.

In the United Kingdom (UK), there is a commonly held assumption that the standard fractionation 70 Gy/35# over 46 days is equivalent to 65 Gy/30# over 39 days when both are administered with synchronous chemotherapy [10,11]. This assumption is evidenced by the use of the six week hypofractionated accelerated schedule with synchronous chemotherapy as the standard arm in randomized studies [12,13]. Using Equations (1) and (2) (*T_p_* = 10 days as both arms employ synchronous chemotherapy), the BEDs and log cell kills of 70 Gy/35# over 46 days and 65 Gy/30# over 39 days are 78 Gy_10_ and 75 Gy_10_ and 10.2 and 9.8. Assuming a dose gradient of 1.2, the modelling would predict a 5% difference in local control. Data from the PET-Neck study appear to show equivalent control rates between the two fractionation schedules when synchronous chemotherapy was delivered [14].

One of the limitations of the modification to the LQ model, where the effect of accelerated repopulation is assumed to be reduced by synchronous chemotherapy, is that the potential effect of synchronous chemotherapy on fraction size sensitivity is ignored. While a significantly less steep dose response gradient in certain head and neck sub-types e.g., Human Papilloma Virus (HPV) related HNSCC may partially explain the apparent equivalence of the UK six week hypofractionated accelerated regime to the standard, the purpose of this paper was to explore simple mathematical solutions where chemotherapy is modelled to effect both fraction size sensitivity and accelerated repopulation.

## 2. Methods

Three assumptions about clinical efficacy of chemoradiation schedules in a hypothetical SCCHN population were made. The first two assumptions use regime comparisons where both fraction size and overall treatment time varied between the comparators.

Firstly, in line with the results of the Radiotherapy and Oncology Group (RTOG) 0129 trial cisplatin chemotherapy delivered with 70 Gy in 35 fractions over 46 days (schedule 1) results in equivalent local control to the same chemotherapy delivered with 36 Gy delivered in 18 fractions followed by 36 Gy delivered in 24 fractions (2 fractions per day) over a total of 39 days (schedule 2) [5]. Secondly in line with the assumption made by UK clinical oncologists discussed in the introduction, cisplatin chemotherapy delivered with 70 Gy in 35 fractions over 46 days results in equivalent local control to the same chemotherapy delivered with 65 Gy in 30 fractions over 39 days (schedule 3).

To express this mathematically the dose loss to accelerated repopulation component of Equation (1) was simplified to give Equation (3).

(3) $$tBED=D\left[1+\left(\frac{d}{\frac{\alpha }{\beta }}\right)\right]-kT$$

where *tBED* = biologically effective dose for tumour (Gy*_α/β_*), *D* = total dose (Gy), *d* = dose per fraction (Gy), *α* = linear component of cell kill (Gy^−1^), *β* = quadratic component cell kill (Gy^−2^), *T* = overall treatment time (days), *k* = dose loss per day (Gy*_α/β_*day^−1^) where accelerated population is assumed to occur equally over the overall treatment time, i.e.:

(4) $$kT=\left[\frac{ln2}{\alpha }\times \frac{\left(T-T_{k}\right)}{T_{p}}\right]$$

This implies a value of k for a standard 7 week course of radiotherapy delivered without synchronous chemotherapy (70 Gy in 35 fractions over 46 days) (*α* = 0.3 Gy^−1^; *T_k_* = 21; *T_p_* = 3 days) of 0.42 Gy_10_day^−1^

The first clinical assumption was then expressed as:

(5) $$tBED_{schedule\;1}=tBED_{schedule\;2}$$

Substituting values for the two schedules gives:

(6) $$70\left[1+\left(\frac{2}{\frac{\alpha }{\beta }}\right)\right]-k46=36\left[1+\left(\frac{2}{\frac{\alpha }{\beta }}\right)\right]+36\left[1+\left(\frac{1.5}{\frac{\alpha }{\beta }}\right)\right]-k39$$

This can be simplified to:

(7) $$\frac{\alpha }{\beta }=\frac{14}{7k+2}.$$

The second clinical assumption was then expressed as:

(8) $$tBED_{schedule\;1}=tBED_{schedule\;3}$$

Substituting values for the two schedules gives:

(9) $$70\left[1+\left(\frac{2}{\frac{\alpha }{\beta }}\right)\right]-k46=65\left[1+\left(\frac{2.17}{\frac{\alpha }{\beta }}\right)\right]-k39$$

which can be simplified to:

(10) $$\frac{\alpha }{\beta }=\frac{1.05}{5-7k}$$

The curves for Equations (5) and (7) were then plotted to determine possible values for *α/β* and *k*.

In order to calculate the value of *α* for potential values of *α/β* and *k*, the third assumption, namely that the log_10_cell kill of 70 Gy in 35 fractions with synchronous cisplatin is approximately equivalent to the log_10_cell kill of 84 Gy in 70 fractions radiation alone (calculated in the introduction to be 9.7) was used based on meta-analysis data [8].

**Figure 1:**
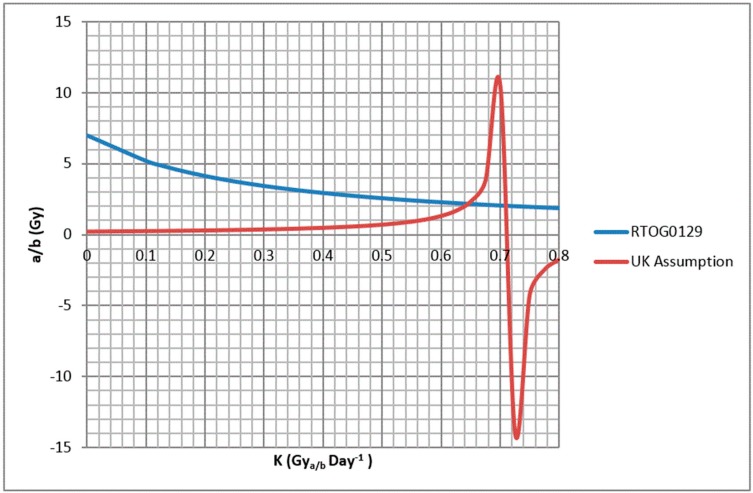
Alpha beta ratio plotted against k for formulas satisfying the assumed equivalent outcomes in the Radiotherapy and Oncology Group (RTOG) 0129 study (blue line) and between 70 Gy in 35# over 46 days with synchronous chemotherapy and 65 GY in 30# over 39 days with synchronous chemotherapy (UK assumption red line).

Based on the potential values of *α/β* and k extracted from the graph in Figure 1, the *tBED* for 70 Gy in 35 fractions with synchronous chemotherapy was first calculated using Equation (3). *α* was then calculated by re-arranging Equation (2) using this *tBED*.

## 3. Results

The curves for Equations (5) and (7) are represented in Figure 1. The curves cross at a value of *α/β* 2 Gy with value of *k* 0.65 Gy_2_day^−1^ or 0.71 Gy_2_day^−1^.

For *α/β* = 2 Gy and *k* = 0.65 Gy_2_day^−1^, *α* = 0.20 Gy^−1^. For *α/β* = 2 Gy and *k* = 0.71 Gy_2_day^−1^, *α* = 0.21 Gy^−1^.

The log cell kill of various chemoradiation fractionation schedules is documented in Table 1 for the above values.

## 4. Discussion

The derivation of values of *α/β* of 2 Gy, *k* approximately 0.7 Gy_2_day^−1^, and *α* approximately 0.2 Gy^−1^ are based on three assumptions: the equivalence of the worldwide standard of 70 Gy in 35 fractions over 46 days with synchronous cisplatin to firstly the RTOG hyperfractionated regime 72 Gy in 42 fractions over 39 days, to secondly the UK hypofractionated regime 65 Gy in 30 fractions over 39 days, and to thirdly the hyperfractionated dose escalated regime 84 Gy in 70 fractions over 46 days without synchronous chemotherapy. The first of these assumptions is based on the outcome of a randomized trial, the third on a meta-analysis and the second is an assumption widely held among UK clinicians participating in randomized trials but also supported by data from the PET-Neck study [5,8,14].

In addition to the assumptions stated above, there are several limitations to such modelling. Firstly, these values are based on equivalence in a hypothetical head and neck population. In reality, they will vary for individual tumours and therefore the mean values of these parameters will likely vary for example between populations of p16+ve oropharyngeal carcinoma, p16-ve oropharyngeal carcinoma, early laryngeal carcinoma and locally advanced laryngeal/hypopharyngeal carcinoma. In addition, the values for patients receiving neoadjuvant chemotherapy are likely to be different.

Despite these limitations, this paper attempts to propose modelling of synchronous cisplatin chemotherapy in HNSCC by alteration of both fraction sensitivity and accelerated repopulation parameters. Such modelling suggests that in the presence of synchronous chemotherapy accelerated repopulation may remain a significant factor and in addition fraction sensitivity may be increased by synchronous chemotherapy. We have not attempted, in this paper, to model the effect of synchronous chemotherapy on parameters related to acute mucositis. For these reasons, continued examination of synchronous chemotherapy with dose escalated hypofractionated accelerated regimes is warranted in appropriate populations within prospective randomized trials. While the use of neoadjuvant chemotherapy will affect the values of the parameters calculated in this paper by reducing clonogen number, it may make any benefit from the increased log cell kill predicted with such regimes difficult to detect in national randomized trials. Similarly, the inclusion of large numbers of patients with small volume good prognosis tumours may also mask any benefit. These regimes should initially be tested therefore in poor prognosis groups in the absence of neoadjuvant chemotherapy such as the ongoing international COMPARE study [20,21].

## 5. Conclusions

The parameters calculated in this paper suggest a continued role for the investigation of hypofractionated accelerated regimens with synchronous chemotherapy provided this is done in an appropriately poor prognosis, locally advanced population with regimes which have been tested prior to sufficiently large phase 3 trials. The assumptions and the limitations of this and all modelling mean this is hypothesis generating only and requires rigorous prospective validation.

## Figures and Tables

**Table 1:** Comparison of log cell kill values predicted for various regimes administered with synchronous chemotherapy using parameters derived in this paper.

Regime	Reference	Total Dose (Gy)	Fractions	Dose Per Fraction (Gy)	Overall Treatment Time (Days)	Log_10_Cell Kill (*α/β* = 2 Gy; *k* = 0.65 Gy_2_day^−1^; *α* = 0.20 Gy^−1^)	Log_10_Cell Kill (*α/β* = 2 Gy; *k* = 0.71 Gy_2_day^−1^; *α* = 0.21 Gy^−1^)
International Standard	[15]	70	35	2.00	46	9.7	9.7
UK Alternative 6 weeks	[12,13,14]	65	30	2.17	39	9.7	9.7
Manchester/Birmingham 4 weeks	[16,17,18,19]	55	20	2.75	25	10.1	10.2
ART-DECO experimental arm 5.5 weeks	[12]	67.2	28	2.40	37	10.9	11.0
COMPARE experimental arm 5 weeks	[20]	64	25	2.56	32	11.0	11.1

## References

[1] Fowler J.F. (1989). A Review: The linear quadratic formula and progress in fractionated radiotherapy. Br. J. Radiol..

[2] Fowler J.F. (2010). 21 years of biologically effective dose. Br. J. Radiol..

[3] Fowler J.F. (2007). Is there an optimum overall time for head and neck radiotherapy? A review, with new modelling. Clin. Oncol..

[4] Fowler J.F. (2008). Optimum overall times II: Extended modelling for head and neck radiotherapy. Clin. Oncol..

[5] Nguyen-Tan P.F., Zhang Q., Ang K.K., Weber R.S., Rosenthal D.I., Soulieres D., Kim H., Silverman C., Raben A., Galloway T.J. (2014). Randomized phase III trial to test accelerated versus standard fractionation in combination with concurrent cisplatin for head and neck carcinomas in the Radiation Therapy Oncology Group 0129 trial: Long-term report of efficacy and toxicity. J. Clin. Oncol..

[6] Bourhis J., Sire C., Graff P., Grégoire V., Maingon P., Calais G., Gery B., Martin L., Alfonsi M., Desprez P. (2012). Concomitant chemoradiotherapy versus acceleration of radiotherapy with or without concomitant chemotherapy in locally advanced head and neck carcinoma (GORTEC 99-02): An open-label phase 3 randomised trial. Lancet Oncol..

[7] Meade S., Sanghera P., McConkey C., Fowler J., Fountzilas G., Glaholm J., Hartley A. (2013). Revising the radiobiological model of synchronous chemotherapy in head-and-neck cancer: A new analysis examining reduced weighting of accelerated repopulation. Int. J. Radiat. Oncol. Biol. Phys..

[8] Lacas B., Bourhis J., Overgaard J., Zhang Q., Grégoire V., Nankivell M., Zackrisson B., Szutkowski Z., Suwiński R., Poulsen M. (2017). Role of radiotherapy fractionation in head and neck cancers (MARCH): An updated meta-analysis. Lancet Oncol..

[9] Hartley A., Sanghera P., Glaholm J., Mehanna H., McConkey C., Fowler J. (2010). Radiobiological modelling of the therapeutic ratio for the addition of synchronous chemotherapy to radiotherapy in locally advanced squamous cell carcinoma of the head and neck. Clin. Oncol..

[10] Bhide S.A., Ahmed M., Barbachano Y., Newbold K., Harrington K.J., Nutting C.M. (2008). Sequential induction chemotherapy followed by radical chemo-radiation in the treatment of locoregionally advanced head-and-neck cancer. Br. J. Cancer.

[11] Loo S.W., Geropantas K., Wilson P., Martin W.M., Roques T.W. (2013). Target volume definition for intensity-modulated radiotherapy after induction chemotherapy and patterns of treatment failure after sequential chemoradiotherapy in locoregionally advanced oropharyngeal squamous cell carcinoma. Clin. Oncol..

[12] Gujral D.M., Miah A.B., Bodla S., Richards T.M., Welsh L., Schick U., Powell C.J., Clark C.H., Bidmead M.A., Grove L. (2014). Final long-term results of a phase I/II study of dose-escalated intensity-modulated radiotherapy for locally advanced laryngo-hypopharyngeal cancers. Oral Oncol..

[13] Thomson D., Yang H., Baines H., Miles E., Bolton S., West C., Slevin N. (2014). NIMRAD—A phase III trial to investigate the use of nimorazole hypoxia modification withintensity-modulated radiotherapy in head and neck cancer. Clin. Oncol..

[14] Hartley A., Fong C., Sanghera P., Wong W.L., McConkey C., Rahman J., Nutting C., Al-Booz H., Robinson M., Junor E. (2016). Radiation therapy variation in the randomized phase 3 positron emission tomography neck study. Int. J. Radiat. Oncol. Biol. Phys..

[15] Forastiere A.A., Zhang Q., Weber R.S., Maor M.H., Goepfert H., Pajak T.F., Morrison W., Glisson B., Trotti A., Ridge J.A. (2013). Long-term results of RTOG 91-11: A comparison of three nonsurgical treatment strategies to preserve the larynx in patients with locally advanced larynx cancer. J. Clin. Oncol..

[16] Benghiat H., Sanghera P., Cashmore J., Hodson J., Mehanna H., Simmons R., Massey P., Sangha G., Bode C., Cooper P. (2014). Four week hypofractionated accelerated intensity modulated radiotherapy and synchronous carboplatin or cetuximab in biologically staged oropharyngeal carcinoma. Cancer Clin. Oncol..

[17] Sanghera P., McConkey C., Ho K.F., Glaholm J., Hartley A. (2007). Hypofractionated accelerated radiotherapy with concurrent chemotherapy for locally advanced squamous cell carcinoma of the head and neck. Int. J. Radiat. Oncol. Biol. Phys..

[18] Jegannathen A., Mais K., Sykes A., Lee L., Yap B., Birzgalis A., Homer J., Ryder W.D., Slevin N. (2011). Synchronous chemoradiotherapy in patients with locally advanced squamous cell carcinoma of the head and neck using capecitabine: A single-centre, open-label, single-group phase II study. Clin. Oncol..

[19] Jegannathen A., Swindell R., Yap B., Lee L., Sykes A., Mais K., Sanghera P., Hartley A., Glaholm J., Slevin N. (2010). Can synchronous chemotherapy be added to accelerated hypofractionated radiotherapy in patients with base of tongue cancer?. Clin. Oncol..

[20] Meade S., Gaunt P., Hartley A., Robinson M., Harrop V., Cashmore J., Wagstaff L., Babrah J., Bowden S.J., Mehanna H. (2018). Feasibility of Dose-escalated Hypofractionated Chemoradiation in Human Papilloma Virus-negative or Smoking-associated Oropharyngeal Cancer. Clin. Oncol..

[21] Fong C., Boon I.S., Boon C.S., Benghiat H., Hickman M., Nightingale P., Hartley A., Sanghera P. (2017). Hypofractionated Accelerated Chemoradiation for Oropharyngeal Cancer and the 2016 Royal College of Radiologists’ Fractionation Guidelines. Clin. Oncol..

